# The open abdomen, indications, management and definitive closure

**DOI:** 10.1186/s13017-015-0026-5

**Published:** 2015-07-25

**Authors:** Federico Coccolini, Walter Biffl, Fausto Catena, Marco Ceresoli, Osvaldo Chiara, Stefania Cimbanassi, Luca Fattori, Ari Leppaniemi, Roberto Manfredi, Giulia Montori, Giovanni Pesenti, Michael Sugrue, Luca Ansaloni

**Affiliations:** General Surgery Department, Papa Giovanni XXIII Hospital, Piazza OMS 1, 24127 Bergamo, Italy; Denver Health Medical Center, Denver, CO USA; General surgery Department, Ospedale Maggiore, Parma, Italy; Niguarda Trauma Center, Ospedale Niguarda Ca’Granda, Milan, Italy; Unità Operativa di Chirurgia d’Urgenza, Azienda Ospedaliera “San Gerardo”, Monza, Italy; Department of Abdominal Surgery, University of Helsinki, Helsinki, Finland; Letterkenny Hospital and the Donegal Clinical Research Academy, Donegal, Ireland; University College Hospital, Galway, Ireland

**Keywords:** Open abdomen, Peritonitis, Pancreatitis, Trauma, Management, Closure

## Abstract

The indications for Open Abdomen (OA) are generally all those situations in which is ongoing the development an intra-abdominal hypertension condition (IAH), in order to prevent the development of abdominal compartmental syndrome (ACS). In fact all those involved in care of a critically ill patient should in the first instance think how to prevent IAH and ACS. In case of ACS goal directed therapy to achieve early opening and early closure is the key: paradigm of closure shifts to combination of therapies including negative pressure wound therapy and dynamic closure, in order to reduce complications and avoid incisional hernia.

There have been huge studies and progress in survival of critically ill trauma and septic surgical patients: this in part has been through the great work of pioneers, scientific societies and their guidelines; however future studies and continued innovation are needed to better understand optimal treatment strategies and to define more clearly the indications, because OA by itself is still a morbid procedure.

## Introduction

The first to describe the use of the open abdomen (OA) technique, in a generalized peritonitis was probably Andrew J. McCosh in 1897 [1]. However this clinical approach to a critically ill patient at that time was unusual and while again referred to by Ogilvie in the mid 1940’s [2] and only recently became popular in patients undergoing damage control surgery (DCS). The indications for Open Abdomen are generally trauma, abdominal sepsis, severe acute pancreatitis and in general situations in which is ongoing the development an intra-abdominal hypertension condition (IAH), in order to prevent the development of abdominal compartmental syndrome (ACS). The concept of abdominal damage control surgery has two basic components; controlling bleeding and contamination in the abdominal cavity, and leaving the abdomen open, to decompress or facilitate return at planned re-laparotomy. Maintaining the abdomen domain requires a temporary abdominal closure (TAC). Unlike in trauma patients with massive bleeding, the main aims of the OA approach both in severe secondary peritonitis and severe acute pancreatitis (SAP) are sepsis control and expedite subsequent surgical interventions.

**Fig. 1:**
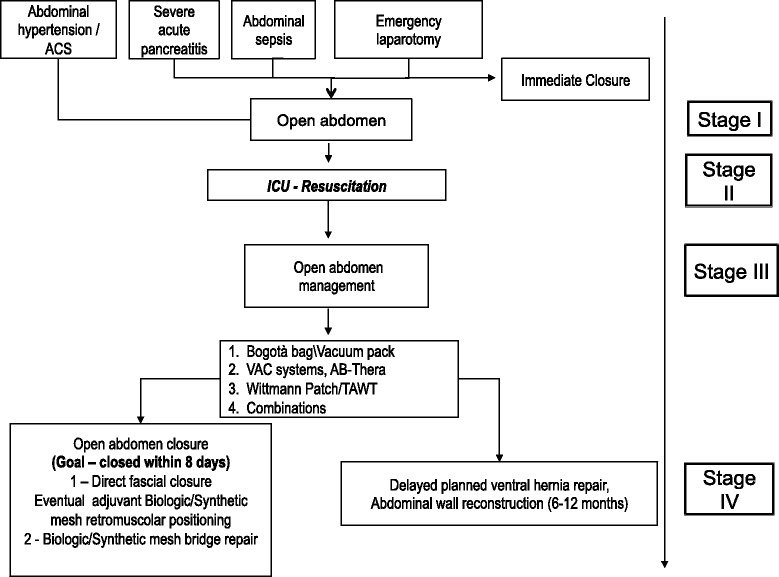
Schematic flow-chart for the treatment of the open abdomen

Mortality rates are high, usually over >30 % [3] depending on the patient cohort. The challenging situation to manage requires a multidisciplinary approach by the surgeon and the ICU team in a specific staged process (Fig. 1).

### Pathophysiology of abdominal compartment syndrome

#### Definitions

While recognized for over a century ACS was returned to clinical care in the 1980’s, when Kron and colleagues [4] described the course of its development following repair of a ruptured abdominal aortic aneurysm. The term was coined by Fietsam in 1989 in patients undergoing abdominal aortic surgery. Since that time, much progress has been made in its management, including the detection, treatment, and prevention [5].

The World Society of the Abdominal Compartment Syndrome convened in 2004 to create a consensus statement on the definition, diagnosis, and treatment of ACS [6]. Table 1 contains a partial list of the definitions. The abdomen and pelvis, while anatomically distinct, represent a single space and thus should be considered as one in discussion of intra-abdominal pressure (IAP) and ACS. The abdominopelvic cavity is a closed space, and the elasticity of its walls and the character of its contents determine the IAP. The IAP is fairly uniform throughout and therefore measurement anywhere within the cavity reflects the entire cavity. The IAP varies with diaphragmatic excursion: it increases with diaphragmatic contraction (inspiration) and decreases with expiration. The abdominal perfusion pressure (APP), analogous to the cerebral perfusion pressure, has been proposed as a more accurate predictor of visceral perfusion and consequently a target for intervention. A target APP of ≥60 mmHg is associated with improved survival in the setting of IAH and ACS [6, 7].

**Table 1:** Consensus definitions related to intra-abdominal hypertension and abdominal compartment syndrome

IAP	The steady-state pressure concealed within the abdominal cavity. Normal = 5–7 mmHg in critically ill adults
APP	MAP – IAP
IAH	Sustained or repeated pathological elevation of IAP ≥12 mmHg
ACS	Sustained IAP >20 mmHg (with or without APP <60 mmHg) that is associated with a new organ dysfunction or failure
Primary ACS	ACS associated with injury or disease in abdomino-pelvic cavity
Secondary ACS	ACS in absence of conditions originating in the abdomino-pelvic cavity

*IAP* intra-abdominal pressure, *APP* abdominal perfusion pressure, *MAP* mean arterial pressure, *IAH* intra-abdominal hypertension, *ACS* abdominal compartment syndrome. Adapted from Malbrain et al. [6]

Normal IAP is actually below 0 mmHg. In the setting of conditions such as morbid obesity, pregnancy, liver disease with ascites, IAP may be chronically elevated to 10–15 mmHg without evidence of altered physiology [6]. During illness or following surgery, IAP is higher, on the order of 5–7 mmHg, due to factors such as tissue edema or ileus. The point at which IAP becomes IAH has been a matter of debate; the consensus definition settled on 12 mmHg as this is the lowest point at which pathologic effects are noted [6]. A grading system was proposed by the Denver General Hospital group in 1996, in the interest of guiding interventions [8]. The WSACS consensus definition varies slightly and is as follows: Grade I (12–15 mmHg); Grade II (16–20 mmHg); Grade III (21–25 mmHg); Grade IV (>25 mmHg) (Table 1) [6].

ACS develops as a result of alterations in perfusion related to IAH. Early literature on the syndrome variably defined the ACS; generally speaking, it was felt that ACS represented a pathologic elevation of IAP that was associated with organ dysfunction. The consensus definition selected a sustained IAP >20 mmHg, recognizing that lower levels of IAH may be associated with organ dysfunction. The final common pathway of organ dysfunction is hypoperfusion. The effects and manifestations of IAH on various organ systems are listed in Table 2 [9].

**Table 2:** The effects of IAH on various organ systems, and clinical manifestations of ACS

System	Effect	Manifestation
*Renal*	Renal vein compression, cortical arteriolar compression	Oliguria, rising creatinine
*Pulmonary*	Upward pressure on diaphragm, decreased compliance and functional residual capacity, increased air way resistance	Hypoxia, hypercarbia, elevated airway pressure, decreased tidal volume
*Cardiovascular*	Decreased venous return, increased afterload	Decreased cardiac output
*Cerebral*	Increased intrathoracic pressure with decreased cerebral venous outflow	Elevated Intra Cranial Pressure
*Splanchnic*	Decreased perfusion of liver and intestine	Metabolic acidosis, bowel ischemia

ACS is classified as primary if it is the result of a pathophysiologic process within the abdominopelvic cavity. It may be a result of bleeding, acute accumulation of ascites fluid, rapidly growing tumor or other mass, retroperitoneal edema, packing of visceral injuries, etc. Secondary ACS refers to development of ACS in the absence of a primary abdominopelvic process. In early descriptions, the secondary ACS was identified in patients who had massive resuscitation from hemorrhage or sepsis [10]. Ischemia/reperfusion injury may leads to massive accumulation of ascites, and bowel and retroperitoneal edema from accumulation of extracellular/extravascular fluid [11]. Increasingly it is recognized that secondary ACS is partly iatrogenic secondary to excessive fluid resuscitation.

#### Diagnosis and treatment

The role of elevated IAP is fundamental in understanding the evolution from IAH to ACS. Basing on the different patients and the setting into which they are admitted the measurement of IAP can be less or more helpful but in any case it remains a cornerstone. Recently Starkopf et al. found that the risk of IAH in mechanically ventilated patients is very low especially if they have a positive end expiratory pressure (PEEP) < 10 cm H2O, PaO2/FiO2 > 300 and BMI < 30 kg/m2 and without pancreatitis, hepatic failure/cirrhosis with ascites, gastrointestinal bleeding or laparotomy and the use of vasopressor/inotropes at admission [12]. In all those patients with the aforementioned characteristics the measurement of IAP might be considered.

According to the World Society of the Abdominal Compartment Syndrome 2013 consensus guidelines, IAP should be measured when there are at least 2 known risk factor for IAH/ACS in critically ill or injured patients [13]. Serial measurements should be performed during the patient’s critical illness, preferably every 4–6 h. To measure the IAP the bladder pressure is considered the gold standard and should be taken at end-expiration with the patient supine and the transducer zeroed at the midaxillary line after an instillation of saline into the bladder. Another option available, if the bladder pressure is contraindicated (due to a constitutive augmented bladder pressure, e.g. in pelvic hematoma), is the measurement with the stomach technique [14]. Moreover exist the possibility to measure the intra-abdominal pressure via rectal, vaginal, inferior vena cava and direct intra-peritoneal measurement.

In treating patients with IAH, key principles include: optimization of systemic perfusion and organ function, institution of specific medical procedures to reduce IAP, and prompt surgical decompressive laparotomy for refractory IAH. Great emphasis has been placed on prevention, with early, prompt haemostatic control, aggressive balancing of resuscitation to include abundant coagulation factors. Tailoring the response for individual patients is essential to ensure that optimal outcomes are achieved. Further measures to alleviate IAH include sedation, analgesia, and neuromuscular blockade can be used to decrease IAP. Is it has been shown that persistent IAH of >18 mmHg is an independent cause of renal failure in general surgical patients admitted to ICU [15].

When employing early goal-directed fluid resuscitation is crucial to remember that correction of hypovolemia must be balanced carefully to avoid an iatrogenic secondary abdominal compartment syndrome. The keys in the end are prompt return to status quo, hemorrhage control, eradication of sepsis, removal of fluid either by percutaneous catheter drainage and finally decompression.

### Open abdomen in trauma

The management of complex problems in major trauma patients using OA and TAC techniques has gained popularity and become a valuable tool for the emergency surgeon. Notwithstanding the evolution of supportive care and the development of new and sophisticated commercial devices for TAC have significantly simplified clinical management, OA is still associated with serious complications such as nutritional problems with fluid and protein loss, loss of abdominal domain secondary to fascial retraction, frozen abdomen and entero-atmospheric fistulas (EAF). For these reasons, OA should be reserved for selected cases only and with the aim of obtaining early abdominal closure, possibly with primary fascial repair [16]. In trauma patients frequent indications for OA after injury are the prevention or treatment of ACS, the need for a “second look” operation in abdominal injuries, post-injury septic abdomen and injury with partial or entire loss of the abdominal wall.

#### Prevention and treatment of IAH/ACS

The prevalence of ACS in trauma patients has fallen in centers with advanced medical care, some reporting falls from 30 to almost 0 % [5]. Where trauma and emergency surgery systems are not so advanced IAH and ACS can be expected to occur in up to 40 % of ICU admission respectively. Risk factors for IAH/ACS in trauma patients are: (i) increased intra-retroperitoneal contents consequent to hemorrhage from organ or pelvic injury, or emergency surgery with packing procedures, (ii) increased intraluminal contents occurring in post-injury bowel paresis, (iii) decreased abdominal wall compliance when abdominal injury occurs in patients with high body mass index or for an associated third degree burn of the abdominal wall and (iv) increased visceral edema following massive fluid/blood resuscitation. Untreated post-injury ACS is an independent predictor of organ failure [17] that is often difficult to reverse, and should be prevented using different strategies. delayed decompression may not reverse the sequalae of IAH and ACS [18]. At the end of a damage control operation, it would be ideal to measure IAP to decide for fascial closure: however this is technically difficult unless a continuous IAP technique is used [5]. Some authors prefer to left open the abdomen for 24–48 h if the value is greater than 12 mmHg Moreover, in trauma patients with elevated IAP (with or without previous surgery) every medical strategy to reduce IAP should be applied: ng tube, colonic decompression, prokinetic medications, supine position, negative fluid balance starting from post-injury day one, percutaneous drainage of fluid collections and sedation and muscle relaxation.

#### Need for a “second look” operation

A second look procedure is planned when the initial operation has been stopped in a damage control setting for physiologic exhaustion of the patient, bleeding from remote areas not amenable to surgical correction, vascular injuries of visceral vessels with risk of bowel ischemia, resected and closed bowel with subsequent need for anastomosis or stoma, complex liver injury treated with packing, and the need for transfer to a higher level facility. All these cases require TAC that allows for a simple re-operation for definitive abdominal care.

#### Post-injury septic abdomen

Septic abdomen may develop following hollow viscus injuries for penetrating or blunt trauma, particularly in cases of delayed diagnosis or leakage after primary repair of colonic wounds. Unusual conditions are septic evolutions of complex duodeno-pancreatic injuries. Recent clinical series suggest that OA associated with negative pressure therapy (NPT) improves observed survival compared with P-POSSUM expected survival in severe peritonitis [19]. In a porcine model of peritoneal fecal contamination, NPT reduced systemic inflammation thereby improving organ function [20].

#### Loss of abdominal wall

This is an unusual condition following penetrating injuries caused by high velocity military weapons or blast injuries. It is sometimes a consequence of extensive surgical debridement after soft tissue infection by necrotizing germs that have spread to the abdominal wall. The abdomen is of necessity open and requires TAC until it is possible to reconstruct the wall.

### Open abdomen in abdominal sepsis and pancreatitis

The main aims of the open abdomen approach in severe secondary peritonitis and severe acute pancreatitis (SAP) are to facilitate the clearance of the infectious material, expedite subsequent surgical interventions and prevent the development of abdominal compartment syndrome (ACS).

#### Abdominal sepsis

In severe secondary peritonitis, a staged approach may be required for three different reasons, although they are often used in combinations.

Firstly, the inability to control the source of contamination in a single operation: Instead of the traditional model of one definitive operation and possible reoperation only performed as needed (relaparotomy on-demand strategy), there are two other options to manage a severely contaminated peritoneal cavity. One, termed planned relaparotomy refers to a technique where the need for a second operation is recognized and decided at the initial operation. Another option, the open abdomen technique, is leaving the abdomen open and treating the infected peritoneal cavity like an “open abscess” with frequent irrigations and TAC techniques [21].

Secondly, if the surgeon feels the patient wont tolerate a definitive repair and/or abdominal wall closure, the operation is deliberately abbreviated due to the severe physiological derangement and suboptimal local conditions for healing, and restoration of intestinal continuity is deferred to the second operation (deferred anastomosis technique) This is particularly important in hypotensive patients who are already received ionotropes [22].

Thirdly, the presence of extensive visceral edema may increase the risk of ACS development, if primary fascial closure is attempted [23]. To prevent ACS, the abdominal incision is left open and the viscera are covered with one of the TAC methods. ACS can develop from a number of complications related to intra-abdominal sepsis including but not limited to large volume fluid resuscitation resulting in visceral edema and intra-abdominal free fluid collection, retroperitoneal, intra-abdominal and abdominal wall bleeding and ileus, pseudo-obstruction and mechanical obstruction of the bowel.

#### Severe acute pancreatitis

Since the late 1970s a treatment option for SAP was an open management with frequent dressing changes in order to facilitate the clearance of infection and an adequate drainage analogously to the open management of an incised abscess.

Patients underwent surgical interventions to achieve control of the infection source, the infected peripancreatic necrosis. If the source control at the initial operation was incomplete and if repeated measures to achieve it were needed, the abdomen was left open between procedures. Gradually the cavity decreased in size and often healed by secondary intention after formation of granulation tissue. Even in the case of an enteric fistula secondary to the open abdomen treatment, expectant management often resulted in acceptable results, although the open management was associated with poorer results than the closed drainage methods [24–27].

It was suggested that a significant proportion of patients with SAP dying of early multiple organ dysfunction syndrome in effect died of unrecognized and untreated ACS caused by massive fluid resuscitation, capillary leak and visceral edema [28, 29].

Although percutaneous drainage of pancreatic ascites can, in some cases, decrease IAP at least temporarily, surgical decompression is the most reliable method to relieve IAH and restore vital organ functions, especially in the pulmonary, cardiovascular and renal systems.

There are three options for surgical decompression in patients with no recent abdominal incision (that often is the case in SAP). A long vertical midline incision is most commonly used and it has been showed to decrease IAP effectively: it is rapid and easy to perform, but it is associated with a risk of intestinal fistulas and in many cases failure to close the fascia requiring complex reconstructive surgery at a later stage [30]. Transverse laparostomy is a promising alternative and isolated reports have shown its effectiveness in reducing IAP [31]. Although it takes slightly longer to perform than midline laparostomy, same principles of managing the open abdomen can be applied without additional equipment.

A third alternative used in SAP is the subcutaneous linea alba fasciotomy, where the fascia is incised through three small skin incisions leaving the rest of the skin and the peritoneum intact [32]. Although it eliminates the OA, it might not be always effective enough [33]. In addition, the subcutaneous fasciotomy always results in a ventral hernia requiring repair later on.

Infected pancreatic necrosis is an established indication for surgical necrosectomy, ideally postponed until 4 weeks after the onset of symptoms and performed most commonly through a midline incision [34]. Because ACS usually commences during the first few days of the disease and the (usually) sterile necrosis is unripe, there are no indications to explore the pancreas or the peripancreatic spaces further. In addition to causing significant bleeding, it could also introduce an infection to the peri-pancreatic space [28]. Although both midline and transverse incisions could later be utilized for necrosectomy, transverse subcostal incision could be justified for decompression when concomitant necrosectomy is planned or anticipated in patients with late onset ACS.

### Management of OA and its definitive closure

The management of patients with OA is a particularly challenging issue that requires compulsorily a multidisciplinary approach with a strength interaction among the surgeon and the intensive care unit (ICU) team in order to offer the best treatment to these critical patients.

#### ICU management

A management plan to reduce risk of ACS developing, both primary and secondary, should ideally begin before the patient gets to the emergency room e from there get to the operatory room where the surgeon decide to leave open the abdomen.

In the ICU a patient with OA requires a specific management. Coagulopathy should be treated with balanced transfusion [35] with a restrictive fluid management strategy in order to prevent acute lung injury (ALI) and acute respiratory distress syndrome (ARDS) [36–39]; in trauma the application of a Exsanguination Protocol with massive transfusion showed a decreased mortality with lower incidence of severe sepsis/septic shock and multi organ failure (MOF) and lower ventilator associated pneumonitis (VAP), ventilatory failure, and ACS [35, 40].

pH should be maintained > 7.2 and checked with a frequent measurement of arterial lactate level. The heat loss in a patient with OA is a problem that should be constantly kept in mind. Hypothermia should be treated reaching an ideal temperature > 37 °C with passive rewarming, air warmers and Bair Hugger Therapy [38].

These patients should receive a tailored ventilatory support with a low tidal volume in order to prevent ALI and ARDS that can be exacerbated by VAP and transfusion-related ALI [41]. Infectious complications, not only in the abdomen, are associated with failed abdominal closure [42, 43]: an adequate antibiotic therapy should be directed toward the underlying disease that culminated in the OA with an empirical anti-enterococcal coverage in patients with prior antibiotic exposure [44]. Another important issue in the ICU is the pain control with a consequent reduction in agitation, stress and fear. Adequate sedation levels should be maintained and strictly monitored in order to reduce the recall of unpleasant experiences and the awareness.

#### Fluid balance and nutrition

Other fundamental issues in critically ill patient with OA are fluid and electrolytes balance and nutrition support. Patients with OA have an increased insensible fluid loss. In addition, these wounds are open into the peritoneal cavity, adding significantly to the amount of fluid loss across the wound surface. Hydration and volume status should be meticulously monitored, ideally the patients weight should be documented daily. The negative pressure wound therapy contributes to a decrease in fluid losses across the open wound surfaces by significantly reducing evaporation [45] and draining into a dedicated canister as V.A.C pack or Ab-thera (KCI, San Antonio, TX), facilitates fluid collection and allows a more accurate estimation of fluid losses from the wound and peritoneal cavity. The measured fluid losses can then be more precisely replaced, and hypovolemia can be minimized or potentially avoided.

Generally a critically ill patient is in a hypercatabolic state that’s associated with muscle proteolysis, acute protein malnutrition, impairment in immune function, and subclinical development of MOF. Moreover OA is a significative source of nitrogen loss in the critically ill patient with an estimate loss of 2 g of nitrogen per liter of abdominal fluid output [46] requiring an adjusted integration. A patient with OA represents one of the sickest, most inflamed, and subsequently most hypermetabolic among surgical patients. A particular attention must be given to this critical aspect: once the resuscitation is near complete and the GI tract allows it enteral nutrition should be initiated as soon as possible, with a clear benefit for the patient in a lower time to fascia closure and a lower pneumonia and fistula rate [47–49].

### Temporary abdominal closure techniques

Several different TAC techniques to left open the abdomen exist. The ideal one should be easy to apply and remove, should allow rapid access to a surgical second-look, should drain secretions, should ease primary closure and should has morbidity and mortality acceptable, should allow easy nursing, and last but not least should be readily available and cheap. During years, different methods for TAC have been proposed. From late ‘70s and during ‘80s, abdominal dressings for OA were quite simple, and the attention during treatment was focused only on protection and control of the bowel outside the abdomen. Through years, the attention of surgeons moved from protection of the ileus to preservation of the peritoneal space and prevention of lateral retraction of the fascia, which are the most important obstacles against the reconstruction of the abdominal wall at the end of the treatment.

One of the simplest and most inexpensive way to cover the viscera is approximating only the skin with a simple running suture or using towel clips. Another easy method is the plastic silo, also known as Bogotà bag, with a non-adherent plastic sheet, usually from sterile 3 lt. urology irrigation bag, sutured between the fascial edges or the skin.

In the 1993 Wittmann described a new technique (Witmann patch, Star Surgical, Burlington, Vt) consisting in two opposite Velcro sheets sutured to the fascia and connected on the middle allowing an easy and fast access to the abdominal cavity, with a simple traction, and a stepwise reapproximation preventing the fascia retraction [50].

**Fig. 2:**
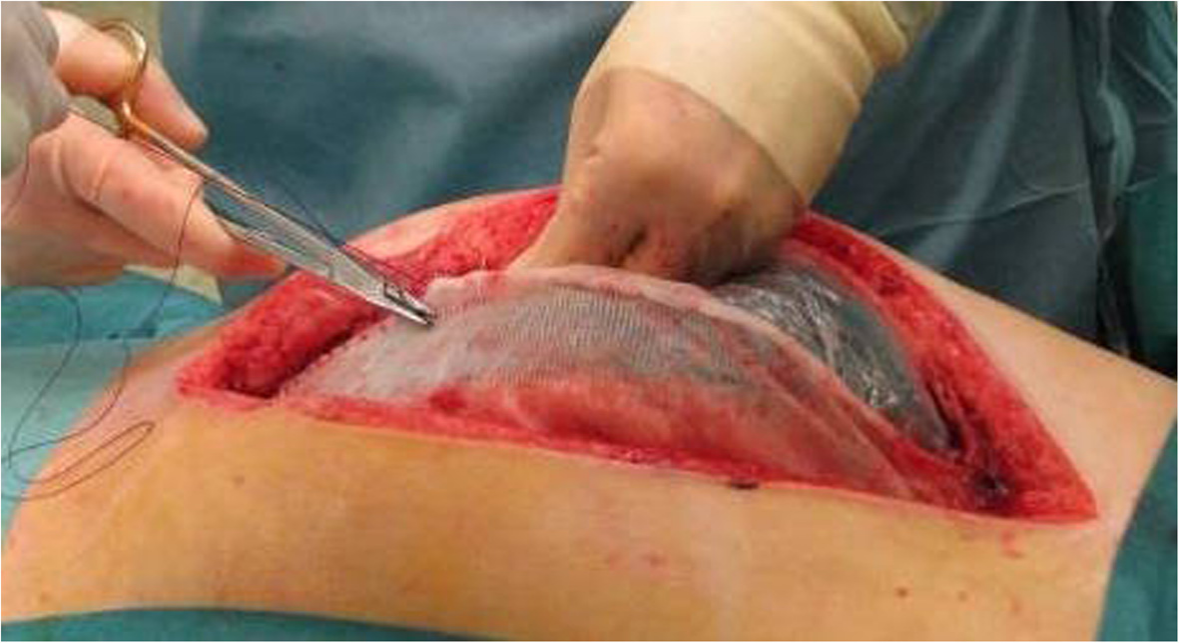
Synthetic mesh sutured to the fascial edges to maintain the traction and prevent the fascial retraction with a plastic sheet posed under in direct contact with the intrabdominal content to protect the bowel

Barker and colleagues in 1995 described another technique, the vacuum pack, where a perforated plastic sheet covers the viscera, sterile surgical towels are placed in the wound, a surgical drain connected with a continue negative pressure is placed on the towels and all is covered by an airtight seal; the dressing should be changed every 2–3 days in operative room but also in the ICU. The negative pressure allows a collection of excess fluid and keeps constant tension on the fascia with a limited cost (50$) [51] with a reported primary fascia closure rate of 68,1 % with a total complication rate of 15 %. [52]. The vacuum pack was then developed with the use of a polyurethane sponge and an adjustable pump to set the negative pressure (KCI V.A.C. Pack, San Antonio, TX) with some advantages as reduced need for frequent dressing changes, increased vascularity of the wound, decreased bacterial counts and extended opportunity for definitive fascial closure (Fig. 2).

Another implementation of the system was introduced by the AB-Thera (KCI, San Antonio, TX) with the use of spider-like sponge that allow a better fluid drainage and a better wound contraction and with a reported primary fascia closure rate of 89 % [53, 54]. When OA is prolonged for more than two days, the AB-Thera system obtains better results [55]. In a prospective observational open-label study involving 20 trauma centers across the USA, was demonstrated that AB-Thera was associated with higher primary fascial closure rate and lower 30 day all-cause mortality, possibly because of the improved peritoneal cytokine removal with this system [55].

A recent modification of the Wittmann patch was described by Dennis et al. in the 2013: the burr like sheet are sutured not directly on the medial fascia but to the underside of the abdominal wall, lateral to the rectus sheath, using external blosters; on the wound is applied a vacuum pack dressing. With this technique they showed a primary fascia closure in 100 % of the patients [56].

Burlew et al. described similar results using the VAC system (KCI, San Antonio, TX) with a polydioxanone (PDS) suture keeping the fascia in a moderate tension and a sequential closure of the abdomen during the following change of dressing, every 2 days [57].

**Fig. 3:**
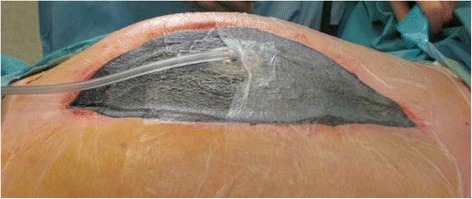
Aspiration system could be placed over the eventual continuous traction system

Acosta described a combined technique using VAC system (KCI, San Antonio, TX) with a polypropylene mesh applied on the fascia edge to keep it in traction and reported a fascia closure rate of 76.6 % [58] (Fig. 3).

Another combined technique consist in the use of the ABRA system (Canica Design Inc, Almonte, Ontario, Canada), which consist in a dynamic fascial tension device with elastomers anchored to the abdominal wall with plastic “button anchors” with the VAC system (KCI, San Antonio, TX) with interesting results and a reported fascial apposition rate of 83 % [59, 60].

Which is the best and the correct management of a patient with OA nowadays is still unclear: the technique is relatively new and in the literature the data and the casuistic reported are too various and too heterogeneous to assess. With these criticisms the techniques seamed to be associated with the best rate of primary closure and minor complication rate are the Negative Pressure Wound therapy and the Wittmann patch but grounded data are needed [61, 62].

### Definitive closure

In the management of OA with TAC the primary goal is to close the wound within 8 days: indeed Miller reported in a large case series, a progressive complication rate increase after the 8th day of OA and increased morbidity and mortality were also reported if the fascia was closed under undue tension [63]. If the primary closure is still impossible to reach the surgeon has different chance. Before the introduction of the temporary closure technique the wounds were closed with, component separation, with granulation tissue with split-thickness skin grafting or with the use of synthetic mesh: in the first case it’s been created a planned ventral hernia that requires a later surgical correction; in the second case the use of a synthetic mesh required a sufficient skin to cover it and exposed the patient to the risk of fistula, adhesion formation and the risk of infection, especially in contaminated fields [64]. A very interesting alternative is the use of Biological Prosthesis (BP) [65, 66]: BP are collagen mesh derived from allogenic or xenogenic sources and they work as a scaffold where the host tissue can growth, covering the wall defect activating a remodelling process in which the host remodels the prosthesis with new healthy tissue. The use of BP gives some advantages: the lowest adhesiogenic potential among the prosthetic material [67], allows blood, growth and pro/anti-inflammatory factors and drugs to reach the surgical field during the healing process enhancing the effect against contamination or infections. They could be divided in cross-linked and non-cross-linked [68, 69]. The crosslinked biological meshes are treated, after the decellularization, in order to obtain crosslinks between and inside the collagen chains: the presence of these bridges prolongs the lengthening of resorption of the mesh ensuring better tensile strength; however, this process slows the fibroblast invasion and angiogenesis, making more difficult the integration with the host tissue and increasing the foreign body response by the prosthesis. The non-crosslinked meshes have a better profile of tissue integration and local inflammatory response, but are subject to a faster resorption process [70]. The Italian Biological Prosthesis Work Group (IBPWG) proposed a decisional model to choose which BP use, cross-linked or not-cross-linked, creating a score on the basis of the presence of infection and the dimension of wall defect. [71] in order to facilitate the decisional process and to obtain the better outcome for the patients.

## Conclusions

All those involved in care of a critically ill patient should in the first instance think how to prevent IAH and ACS. In case of ACS goal directed therapy to achieve early opening and early closure is the key: paradigm of closure shifts to combination of therapies including NPWT and dynamic closure, in order to reduce complications and avoid incisional hernia.

There have been huge studies and progress in survival of critically ill trauma and septic surgical patients: this in part has been through the great work of pioneer and scientific societies, as the WSACS and their guidelines; however future studies and continued innovation are needed to better understand optimal treatment strategies and to define more clearly the indications, because OA by itself is still a morbid procedure.

## Consensus statement

Written informed consent was obtained from the patient for the publication of the images.
